# Protein Binding Nanoparticles as an Integrated Platform for Cancer Diagnosis and Treatment

**DOI:** 10.1002/advs.202202453

**Published:** 2022-08-18

**Authors:** Xuemei Wang, Shengbo Li, Siqi Wang, Shuo Zheng, Zhenbing Chen, Heng Song

**Affiliations:** ^1^ College of Chemistry and Molecular Science Key Laboratory of Combinatorial Biosynthesis and Drug Discovery Wuhan University Wuhan 430072 China; ^2^ Department of Hand Surgery Union Hospital Tongji Medical College Huazhong University of Science and Technology Wuhan 430022 China

**Keywords:** immunotherapy multimodal tumor therapy, multimodal imaging, polydopamine, protein‐binding nanoparticles

## Abstract

Smart nanomaterials constitute a new approach toward safer and more effective combined anti‐cancer immunotherapy. In this study, polydopamine‐multiprotein conjugates (DmPCs) that can be used for targeted delivery of multiple proteins to cells, realize imaging and combine the advantages of multiple treatment methods (photothermal therapy, chemodynamic therapy, and immunotherapy) can be synthesized and characterized. Proteins, as biological agents, are frequently used in this context, given their low toxicity in vivo. To overcome protein instability and short half‐life in vivo, the use of several proteins in combination with selected nanomaterials to treat patients with melanoma is proposed. In addition to the synthesis and characterization of protein‐bound nanoparticles, it is further demonstrated that several proteins can be efficiently delivered to tumor sites. DmPCs have a wide range of potential adaptability, which provides new opportunities for proteins in the field of treatment and imaging.

## Graphical abstract

A novel imaging‐guided multi‐protein‐binding nanomaterial system with the advantages of multiple treatment methods (photothermal therapy, chemodynamic therapy, and immunotherapy) is developed. In this system, the multiple functional proteins are efficiently transported to the tumor site via intravenous injection that performs effectively multimodal cancer therapy with biocompatible nanomaterials.

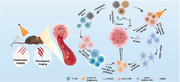

## 1 Introduction

Anti‐cancer immunotherapy is a new treatment approach that acts by stimulating the immune system of patients. Immunotherapy approaches include immune checkpoint blockade (ICB), chimeric antigen receptor T cell engineering therapy ^[^ ^1^ ^]^, and tumor‐targeting vaccines.^[^ ^2^ ^]^ Immunotherapy approaches have been shown to promote significant improvement in the prognostic of a wide range of cancers. Programmed cell death‐1 (PD‐1), a down regulator of immune response, is expressed in activated T and B cells. PD‐1 ligand (PD‐L1) is a transmembrane protein, frequently overexpressed in the surface of tumor cells.^[^ ^3^ ^]^ It has been reported that blocking PD‐1/PD‐L1 interaction can promote tumor recognition and targeting by activated T cells and enhance antitumor immunity, thus being widely used in clinic.^[^ ^4^, ^5^, ^6^ ^]^ Anti‐PD‐1 blocks the PD‐1/PD‐L1 pathway, activates cytotoxic T cells, and stimulates immune response. However, single immunotherapy is often insufficient to treat patients, given that it promotes due to low immune response activation. This may result from poor tumor penetration of therapeutic agents, and from complex tumor immunosuppressive microenvironment,^[^ ^7^ ^]^ thus failing to elicit adequate responses in patients with non‐immunogenic tumors. Immunotherapy regimens that integrate distinct immune system activating approaches, such as immunotherapy combined with photothermal (PTT) or chemodynamic therapy (CDT) have shown significant advances toward tumor eradication.^[^ ^7^, ^8^, ^16^ ^]^ In this context, a synergistic effect has been demonstrated between PTT using nanodrugs and ICB agents. This combined therapy resulted in immune system activation with minimal side effects, leading to efficient tumor depletion in different types of cancer.^[^ ^8^, ^9^, ^10^, ^11^ ^]^ The combination immunotherapy with CDT might enhance the therapeutic action of single immunotherapy, as CDT agents act by boosting the production of the highly oxidized hydroxyl radical (**·**OH), from endogenous hydrogen peroxide (H_2_O_2_), via Fenton reaction, which is key in iron metabolism. Besides the key role of iron in Fenton reaction, the iron ions also accompany depletion of glutathione (GSH), eventually leading to iron toxicity.^[^ ^12^, ^13^, ^14^, ^15^ ^]^ A wide range of nanomaterials enabling tumor imaging have been recently developed.^[^ ^16^, ^17^ ^]^ However, an integrated nanosystem that combines immunotherapy, PTT, CDT, is still lacking. Such system would likely act on a variety of targets at the same time, thus enhancing the potential for a synergistic action.

Proteins have emerged as biological therapeutic agents, that have been used for disease treatment and imaging.^[^ ^18^, ^19^ ^]^ However, due to protein complex structure, hydrophilic nature, surface charge, variability, and easy decomposition in vivo, nanomaterials are required for the active and efficient delivery of proteins into specific cells. Protein‐bound nanoparticles are a novel class of nanoparticles that have been described as safer and efficient in immunotherapy applications. Such nanoparticles have demonstrated excellent biocompatibility and biological stability. The generation of protein‐bound nanoparticles with high functional and structural diversity is expected to be used in drug delivery, enabling tumor imaging and immunotherapy.^[^ ^20^, ^21^ ^]^

Polydopamine (PDA) is stable in aqueous environments, and exhibits good biocompatibility, thus being used as a platform for protein immobilization.^[^ ^22^, ^23^ ^]^ Moreover, polydopamine has excellent photothermal properties and can target the tumor environment with responsive to the weakly acidic condition and the high concentration of GSH.^[^ ^24^, ^25^, ^26^ ^]^ Thus, polydopamine has been suggested as a potential nanoplatform for functional protein immobilization in the context of combination PTT treatment.^[^ ^24^, ^25^ ^]^ Liposome formulations have been successfully used as nanocarriers for passive and active targeted delivery.^[^ ^27^, ^28^, ^29^ ^]^ Using liposome encapsulation, nanoparticles can be selectively adsorbed in target tissues, and proteins can be protected and efficiently transported to the target site, at effective concentration.^[^ ^30^, ^31^, ^32^, ^33^ ^]^

**Scheme 1:**
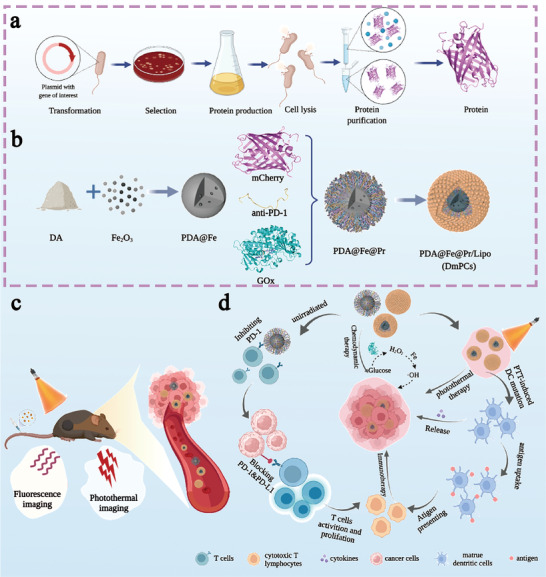
Schematic diagram of DmPCs fabrication and tumor treatment. a) Protein overexpression and purification from bacteria. b) The design and preparation of DmPCs. c) DmPCs targeted tumor and multimodal imaging. d) Schematic diagram of therapeutic mechanism of DmPCs for synergistic self enhancement CDT, photothermal and immunotherapy. The image was created with BioRender.com

In this paper, core–shell protein‐bound nanoparticles with potential application in combined immunotherapy were developed. The designed nanoparticles showed multifunctional properties as anti‐tumor agents, including controlled release of anti‐PD‐1, induction of glucose oxidase (GOx) stimulated CDT, conduction of polydopamine‐based PTT, and fluorescence image‐based tracking of the drug delivery process with protein mCherry. Protein‐bound nanoparticles were constructed using polydopamine‐coated iron nanoparticle as the core for protein loading and surface‐encapsulated liposome as the shell (**Scheme** **1**). With this structural design, the protein content of nanoparticles is increased, when compared to electrostatically adsorbed protein nanoparticles. Moreover, the liposome coated surface helps to stabilize the protein structure and function, as well as to reduce non‐specific cellular uptake. Once concentrated at tumor sites, after intravenous injection, nanoparticles formed by protein grafting onto polydopamine nanoparticles containing Fe (PDA@Fe@Pr) decompose in response to weak acidic conditions and high GSH concentration in the tumor microenvironment. This leads to the release of anti‐PD‐1, GOx and mCherry proteins, as well as of the iron ions loaded inside PDA. Anti‐PD‐1 directly targets tumor cells, thus promoting their recognition and targeting by activated T cells. Subsequently, GOx converts glucose to hydrogen peroxide and gluconic acid, increasing the concentration of hydrogen peroxide, which in turn is key in iron‐catalyzed CDT.^[^ ^34^, ^35^ ^]^ Moreover, glucose consumption by GOx results leads to starvation of cancer cells.^[^ ^36^, ^37^, ^38^ ^]^ Finally, PDA, which exhibits excellent photothermal performance under laser‐irradiation, that is, converts light energy into heat energy, resulting in temperature increase, will further destroy the primary tumor. The presence of mCherry protein allows for the following fluorescence imaging of DmPCs. The system not only provides fluorescence imaging, but also photothermal imaging of the tumor site under laser irradiation. Importantly, these protein‐bound nanoparticles are entirely composed of biocompatible components, therefore showing low cytotoxicity. We propose that our newly generated nanoparticles have therapeutic and imaging value, associated with mild side effects, thus being interesting candidates in the context of combined immunotherapy agents.

## 2 Results and Discussion

### 2.1 Nanoparticle Synthesis and Characterization

**Figure 1:**
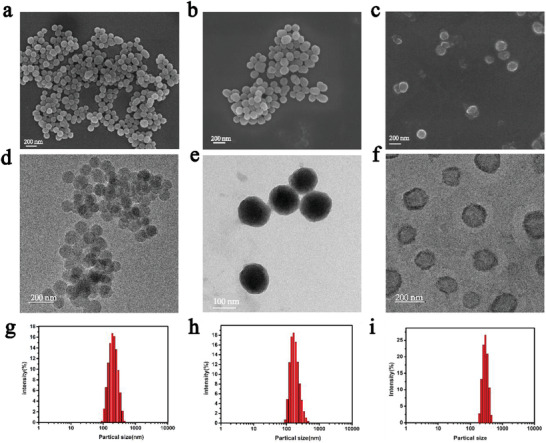
The SEM images of a) PDA@Fe, b) PDA@Fe@Pr, c) DmPCs. The TEM images of d) PDA@Fe, e) PDA@Fe@Pr, f) DmPCs and the size distribution of g) PDA@Fe, h) PDA@Fe@Pr, i) DmPCs.

**Figure 2:**
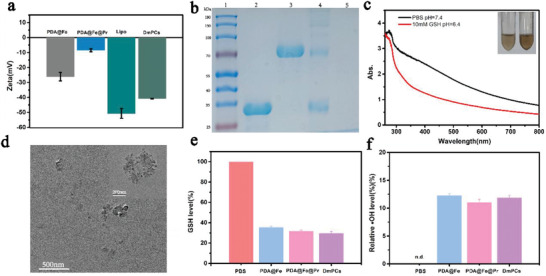
a) Zeta potential of PDA@Fe, PDA@Fe@Pr, Lipo, and DmPCs. b) SDS‐PAGE gel of mCherry and GOx from the protein; Lane 1: Sigma low‐range marker; Lane 2: mCherry; Lane 3: GOx; Lane 4: DmPCs; Lane 5: PDA@Fe. c) UV−vis absorption spectra of DmPCs solution after incubation in different conditions for 72 h. d) The TEM image of DmPCs under 10 mm GSH conditions at 37 °C. e) GSH levels treated under PBS, PDA@Fe, PDA@Fe@Pr, and DmPCs. f) ^•^OH levels treated under PBS, PDA@Fe, PDA@Fe@Pr, and DmPCs.

First, polydopamine coated iron (PDA@Fe) with an average diameter of 107 nm was synthesized (**Figure** **1**a,d,g, and Figure S1a,b, Supporting Information). Then, anti‐PD‐1, mCherry, and GOx were stably immobilized on the surface of PDA@Fe by esterification reaction to form a core–shell structure (Figure 1e and Figure S1c, Supporting Information). As shown in Figure S1b, Supporting Information, transmission electron microscopy (TEM) photographs show the successful preparation of PDA@Fe@Pr with a thin protein shell on the surface. Finally, the liposome shell was coated on the surface, thus enhancing nanoparticle water solubility and improving their circulation time in vivo. Liposome encapsulation PDA@Fe@Pr formed PDA@Fe@Pr/Lipo (DmPCs) are uniformly dispersed nanoparticles with a particle size of around 120 nm analyzed by scanning electron microscope (SEM) and TEM images (Figure 1c,f and Figure S1d, Supporting Information). The DmPCs were analyzed by dynamic light scattering with a hydrated particle size of 180 nm (Figure 1i). X‐ray photoelectron spectroscopy (XPS) showed characteristic peaks representing iron, and TEM energy dispersive spectrometer with iron peaks indicated that iron was successfully loaded into PDA (Figure S2, Supporting Information). Iron content quantification was performed by the phenanthroline colorimetric method, having been calculated as 33.33% by the standard curve (Figure S3, Supporting Information). Compared with PDA@Fe, the surface‐grafted protein nanoparticles, the negative surface potential was reduced (**Figure** **2**a). To study protein‐nanoparticle binding, we selected three fluorescent proteins (GFP, mCherry, and UnaG), and assessed their potential to establish chemical and electrostatic bonds with the nanoparticle structure. We observed that nanoparticles chemically bound to the three fluorescent proteins displayed three fluorophores. Conversely, electrostatically bound nanoparticles were virtually non‐fluorescent (Figure S4, Supporting Information). To test the encapsulation efficiency of liposomes, we performed centrifugation, obtaining an encapsulation efficiency of 75.17% (Figure S5, Supporting Information). UV–vis spectroscopy showed that DmPCs had absorption peak after 700 nm, revealed that nanomaterial with near‐infrared absorption was successfully constructed (Figure S6, Supporting Information). We detected the presence of proteins by SDA‐PAGE gel electrophoresis (Figure 2b). After 1 week of incubation in PBS (pH = 7.4), no significant aggregates or size changes of DmPCs were observed, suggesting that nanoparticles are stable in aqueous environments, therefore ensuring drug‐loading during blood flow circulation (Figure S7, Supporting Information).

### 2.2 In Vitro Degradation of PDA and Release of Fe

To examine the responsiveness of DmPCs to GSH, DmPCs were dispersed in PBS containing 10 mm of GSH. DmPCs degradation was assessed and compared to DmPCs dispersed in PBS alone. Using UV–vis absorption spectroscopy, we observed that the absorption of DmPCs was reduced in the imitated tumor microenvironment (10 mm GSH and pH 6.5 PBS) compared to control which imitated physiological (pH 7.4 PBS) after 72 h, indicating the decomposition of the DmPCs, together with the discoloration of the sample solution (Figure 2c). TEM and SEM imaging further supported the reduction of nanoparticle content and the intracellular breakdown of DmPCs (Figure 2d and Figure S8a, Supporting Information). Using UV–vis absorption spectroscopy, we observed that DmPCs degraded less in a mildly acidic environment compared to GSH‐containing environment (Figure S8b, Supporting Information). To study the iron release from DmPCs, we have conducted a degradation release test in vitro. As shown in Figure S9, Supporting Information, iron ions were released in the weakly acidic, GSH‐containing solution, indicating that nanoparticles were degraded and released their contents under mildly acidic and certain concentrations of GSH conditions, similar to tumor microenvironment. As a comparison, the control group, that in PBS solution at pH 7.4 showed no release of Fe, further showing that the nanoplatform is stable under normal physiological conditions. Therefore, we propose that DmPCs are decomposed in response to the weakly acidic condition and GSH, which mimic the conditions of tumor microenvironments. In addition, the laser‐induced photothermal effect at 660 nm might also promote the decomposition of PDA and the release of Fe.^[^ ^39^ ^]^ PDA@Fe (0.5 mg mL^−1^) solution were irradiated with 660 nm laser (0.5 W cm^−2^) for 5 min and then characterized by SEM (Figure S10, Supporting Information). The results exhibited the slight decomposition of the nanoparticle surface after light exposure, indicating PTT effect also promotes dopamine nanospheres degradation.

### 2.3 Enzymatic Cascade Activity of DmPCs Nanocomposites

We evaluated the ability of DmPCs to catalyze the production of H_2_O_2_ from glucose during glycolysis. The concentration of H_2_O_2_ was quantified by the reaction of titanium sulfate Ti(SO_4_)_2_ with H_2_O_2_ to produce H_2_TiO_4_, which has a characteristic absorption peak at 405 nm. By monitoring the absorption intensity of H_2_TiO_4_ obtained at 405 nm it was found that both DmPCs and free GOx introduced H_2_TiO_4_, indicating the formation of H_2_O_2_ (Figure S11a, Supporting Information). And the H_2_O_2_ produced gradually increased with time (Figure S11b, Supporting Information). It shows that the DmPCs nanocomposite can catalyze the oxidation of glucose to produce H_2_O_2_.

The functional properties of DmPCs against cancer cells are also partly attributed to the presence of Fe, which is toxic to tumor cells, via radical production. Fe^3+^ is reduced by GSH to Fe^2+^, which consumes GSH and leads to H_2_O_2_ conversion to ^•^OH via the Fenton reaction. To test the ability of Fe from DmPCs in solution to undergo Fenton reaction in a GSH environment, thus causing ferroptosis, we evaluated the GSH consumption of DmPCs, as well as the ^•^OH production. The aqueous solution containing DmPCs in the presence of 6.14 mm GSH showed a 72% depletion of GSH and 10% ^•^OH increased production in the presence of 20 mm H_2_O_2_, compared to the PBS control under the same conditions, indicating that the released Fe from DmPCs can deplete GSH and participate in the Fenton reaction (Figure 2e,f). Figure 2f shows that DmPCs have peroxidase activity and can catalyze the production of ^•^OH from H_2_O_2_. The Fenton reaction produces ^•^OH, which can be detected by evolutionary power reactors (EPR). DMPO was used to capture the ^•^OH produced. A four‐line peak of DMPO‐^•^OH in water was detected in the DmPCs/DMPO system as shown in Figure S11c, Supporting Information.

### 2.4 Evaluation of the Photothermal Properties of DmPCs

**Figure 3:**
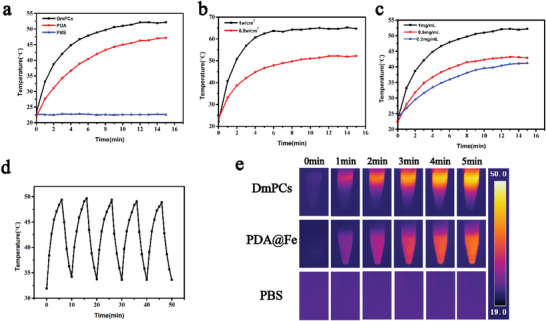
a) Temperature elevation of DmPCs (1 mg mL^−1^), PDA@Fe (1 mg mL^−1^), and PBS under 660 nm laser (0.5 W cm^−2^). b) Temperature curve of DmPCs (1 mg mL^−1^) under different laser power irradiation. c) Temperature changes of DmPCs at different concentrations in 5 min under 660 nm laser (0.5 W cm^−2^) irradiation. d) Photothermal stability of DmPCs (1 mg mL^−1^) in five illumination/cooling cycles. e) Infrared thermographic images of DmPCs (1 mg mL^−1^), PDA@Fe (1 mg mL^−1^), and PBS under 660 nm laser (1.0 W cm^−2^) irradiation for 5 min.

To test the photothermal properties of DmPCs and PDA@Fe, nanoparticles in PBS were irradiated for a period of time with a 660 nm laser (0.5 W cm^−2^), and the resulting temperature changes were recorded accordingly. PBS alone was used as control. As shown in **Figure** **3**a,e, the temperature of DmPCs and PDA@Fe increased from 23 to 52 °C and 47 °C, respectively, whereas the temperature of the PBS control remained at 23 °C. This suggests that the photothermal conversion capacity of DmPCs is superior to that of PDA@Fe, likely resulting from higher water solubility of the first. Figure 3b,c further shows that the DmPCs temperature increases in a concentration and irradiation intensity‐dependent manner, with higher temperatures observed for highly concentrated DmPCs, which are exposed to irradiation for a longer time period. As shown in Figure 3d, the conversion efficiency of DmPCs remains stable after five consecutive cycles of photothermal heating and natural cooling, thus supporting the stability of DmPCs, and their potential use as a photothermal therapeutic agent for the following biomedical applications. The photothermal conversion efficiency (*η*) of the DmPCs was calculated to be 30.88% (Figure S12, Supporting Information).

### 2.5 Evaluation of the Cytotoxicity and GSH Depletion of DmPCs in B16 Cells

**Figure 4:**
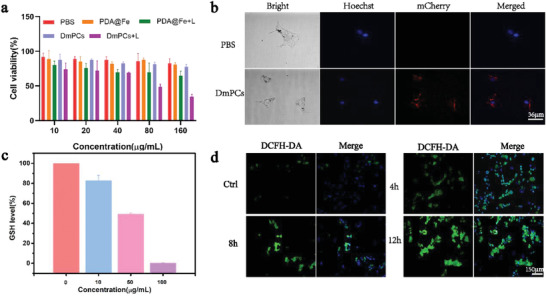
a) Cell viability of B16 cancer cells incubated with diverse concentrations of PDA@Fe and DmPCs. The cells were irradiated with or without a laser (660 nm, 0.5 W cm^−2^, 5 min), followed by further incubation for 24 h. Cytotoxicity test of polydopamine globulin grafted protein. b) Fluorescence images of B16 cells co‐stained with calcein Hoeches under different treatments for 6 h. (Scale bars:36 µm). c) Intracellular GSH in B16 cells treated with DmPCs at different concentrations. d) ROS intensity in B16 cells treated with DmPCs at different time points as examined by fluorescence microscope. (Scale bars:150 µm)

To evaluate the cytotoxicity of DmPCs nanoparticles, we incubated B16 cells with DmPCs or PDA@Fe at different concentrations (10, 20, 40, 80, and 160 µg mL^−1^) for 24 h nanoparticles and exposed to laser irradiation. Then, cell viability was assessed using the cell counting kit 8 (CCK‐8) assay. We observed that, compared to control (PBS), B16 cells incubated with DmPCs alone showed more than 80% viability. In contrast, after cell exposure to laser irradiation, the cell survival rates were significantly decreased, down to 34%, compared with the laser‐shielded cells exposed at the same nanoparticle concentration (**Figure** **4**a). We further evaluated the effect of irradiation on the viability of B16 cells. Our result indicated that the irradiation only revealed no toxic effect. The results showed that the decrease in B16 survival was mainly due to the phototherapy effect of DmPCs under laser irradiation. The same experimental approach was performed using fibrocyte cells to assess the toxicity of DmPCs against normal cells. We observed that normal cells treated with DmPCs and non‐illuminated showed more than 80% viability. Taken together, our results show that DmPCs nanoparticles can efficiently induce cell death in B16 cells with laser irradiation, while maintaining low cytotoxicity against normal cells (Figure S13a, Supporting Information).

In the cytotoxicity test experiments, we also noticed that B16 cells treated with DmPCs and non‐illuminated showed more than 80% viability. We speculate one of the reasons might be that the limited concentration of GSH in the cell culture condition is not enough to effectively break down the PDA to release iron ions. The CDT response is largely dependent on iron ions encapsulated in dopamine nanospheres, but the GSH levels of B16 cells in our culture condition (Table S1, Supporting Information) are much lower than that of tumor sites (≈10 mm).^[^ ^24^ ^]^ Hence, we first verified the intracellular GSH consumption of B16 cells in our culture condition by DmPCs in the absence of laser irradiation. After incubation with different concentrations of DmPCs, the intracellular GSH levels of B16 cells gradually decreased as the concentration of DmPCs increased (Figure 4c), indicating that DmPCs can decrease the GSH content under our cell culture condition. As a comparison, the GSH concentration at tumor sites is ≈10 mm.^[^ ^24^ ^]^ The decomposition of PDA at imitated tumor microenvironment was also conducted and characterized by SEM. The result exhibited that PDA nanospheres was completely destroyed (Figure 2d), indicating that the GSH content under the condition of tumor tissues may effectively decompose PDA. Therefore, the difference in GSH content between in vitro and in vivo cancer cells may cause different Fe release from DmPCs for the CDT response without illumination. In addition, under non‐illuminated condition, there is also a lack of PTT effect that promotes PDA degradation for more Fe release (Figure S10, Supporting Information). These reasons may lead to the little difference of the toxicity experimental results between fibroblast cells and B16 cells in the absence of laser irradiation.

### 2.6 In Vitro Imaging of Composite Nanoparticles

To further demonstrate that DmPCs are effectively delivered to cancer cells, we studied the internalization of nanoparticles in B16 cells after 4 h incubation, by laser confocal microscopy (CLSM). The red fluorescence of mCherry, loaded in DmPCs, was acquired as a measure of the delivery efficiency of the nanoparticles into the cells. Figure 4b shows mCherry fluorescence distributed in the perinuclear region, indicating that DmPCs can effectively permeate the cell membrane and be absorbed by cells. Therefore, the protein, dopamine, and iron ions carried by DmPCs can be effectively delivered to cancer cells with potential therapeutic and imaging functions.

The in vitro CDT potential of DmPCs was determined by the quantification of reactive oxygen species (ROS), using 2′, 7′‐dichlorodihydrofluorescein diacetate (DCFH‐DA). The oxidation of DCFH‐DA by ROS generates green fluorescence, which is proportional to the ROS level. We incubated B16 cells with PDA@Fe/DmPCs nanoparticles at 200 µg mL^−1^ for different time points, after which fluorescence was measured. The results showed that the strongest ROS generation was observed in B16 cells after 12 h of culture (Figure 4d). Comparison between the green fluorescence intensity of cells cultured with PDA@Fe and DmPCs indicated that GOx protein, loaded in DmPCs, induces the production of the radical ^•^OH resulting in significantly higher intensity from cells cultured with DmPCs (Figure S14, Supporting Information).

### 2.7 In Vivo Antitumor Properties of DmPCs

**Figure 5:**
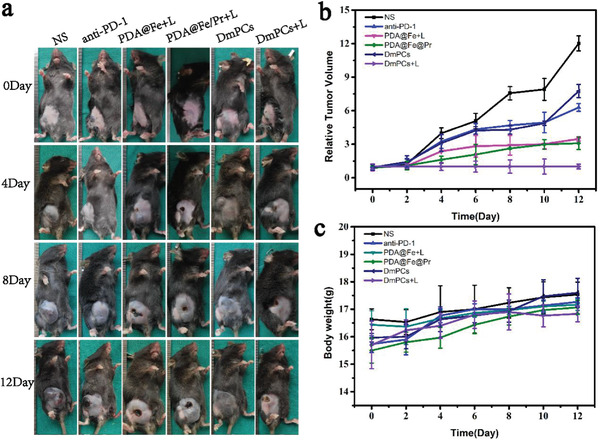
B16 melanoma model. a) Representative digital photos of B16‐tumor‐bearing mice after different treatments. b) Tumor growth curves after various treatments. Data are presented as means ± s.d. (*n* = 6). c) Body weight changes after various treatments. Data are presented as means ± s.d. (*n* = 6).

To investigate the tumor suppressor activities of DmPCs in vivo, we measured total body weight and tumor volume in vivo, using a B16 tumor xenograft model in C57 mice. When the tumor volume in mice increased to ≈50 mm^3^, mice were randomly divided into six groups. Then, 100 µL of saline, anti‐PD‐1, PDA@Fe, PDA@Fe@Pr, and DmPCs, all at a concentration of 150 µg mL^−1^, were injected intravenously every 2 days. Moreover, the PDA@Fe and PDA@Fe@Pr groups were submitted to laser irradiation, whereas DmPCs‐treated mice were with or without laser irradiation, respectively. In the control group, the tumors exhibited rapid growth, as shown in **Figure** **5**a,b. PDA@Fe+L (pink line) and PDA@Fe@Pr+L (green line) showed mild inhibitory effect in tumor growth, compared to the treatment with saline‐treated group (black line). We propose that these observations result from the fact that PTT was performed under laser irradiation. DmPCs (navy line) showed a similar to anti‐PD‐1 (blue line), thus showing the delivery of anti‐PD‐1 to the tumor site. The antitumor effect of DmPCs+L (purple line) was significantly higher than that of PDA@Fe+L and PDA@Fe@Pr+L, indicating that the delivery strategy for therapeutic proteins by DmPCs was effective. The antitumor effect of DmPCs+L was also significantly increased, compared to that of DmPCs‐treated groups (Figure 5b), translating the synergistic effect of PTT, CDT, and immunotherapy. Therefore, tumor growth in mice treated with DmPCs and exposed to laser irradiation was significantly inhibited due to the photothermal effect as well as the immunotherapy and CDT. The results are supported by imaging observations that showed tumor growth in the different treatment groups (Figure 5b). Mice body weight was also examined, and the results indicated no significant differences between the six groups (Figure 5c). All animals were alive after treatments, demonstrating the biosafety of DmPCs under laser irradiation.

**Figure 6:**
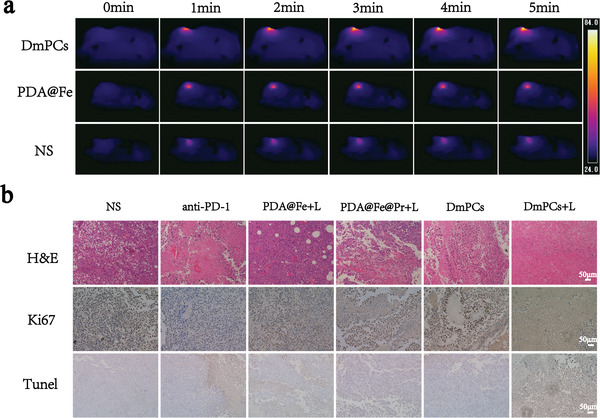
a) Thermal IR images of tumor‐bearing mice (under 660 nm photoirradiation, 0.5 W cm^−2^, 5 min) that were respectively injected with different formulations. b) H&E staining Ki67 staining and TUNEL staining of tumors in various formulations after 12 days of treatments (scale bars: 50 µm).

To confirm these results, hematoxylin and eosin (H&E) staining, terminal deoxynucleotidyl transferase‐mediated dUTP biotin nick end labeling (TUNEL), and Ki‐67 staining were performed in tumor sections. Pathological analysis and examination of the residual tumor areas by H&E revealed that the tumors in the PDA@Fe+L, PDA@Fe@Pr+L, and DmPCs groups exhibited poorly differentiated cancer cells with enlarged and deeply stained nuclei and diffuse distribution of cancer cells with occasional ulceration. In contrast, tumors in the DmPCs+light group exhibited extensive necrosis. The frequency of Ki‐67^+^ proliferating cells was similar in the residual tumor areas of the PDA@Fe+L, PDA@Fe@Pr+L, and DmPCs groups, and significantly lower than in the normal saline (NS) group, with the DmPCs+L group showing the minimum Ki‐67^+^ cells among all groups. Apoptotic cells (TUNEL) in the DmPCs+L group were significantly more frequent than in the other treatment groups (**Figure** **6**b).

### 2.8 In Vivo Photothermal Imaging and Fluorescence Imaging

To evaluate the photothermal properties of DmPCs in vivo, mice were intravenously injected with 100 µg mL^−1^ of PDA@Fe, DmPCs, or saline, respectively. The temperature at the tumor site was monitored and recorded by a thermal imager for comparison. Six hours after intravenous injection, the tumor site was exposed to 0.5 W cm^−2^ 660 nm laser, and temperature changes were detected with a thermal imaging camera. As shown in Figure 6a, in saline‐injected mice, tumor temperature increased from 29 to 39.6 °C. In contrast, in PDA@Fe‐injected mice, tumor temperature increased from 29 to 50 °C. Notably, in mice injected with DmPCs, the temperature at the tumor site increased from 29 °C up to 89 °C, under laser irradiation, therefore confirming the enhanced photothermal conversion capacity of DmPCs, compared to that of PDA@Fe nanoparticles. In addition, we also observed that, during laser irradiation, tumor temperature increases rapidly, indicating that nanoparticles perform photothermal conversion in vivo in a short timeframe, thus showing great potential in antitumor treatment.

DmPCs were injected into B16 tumor‐bearing mice for observation. As shown in Figure S15, Supporting Information, fluorescence of mCherry was detected in the tumor region of the mice after 8 h.

### 2.9 In Vivo Immune Response

**Figure 7:**
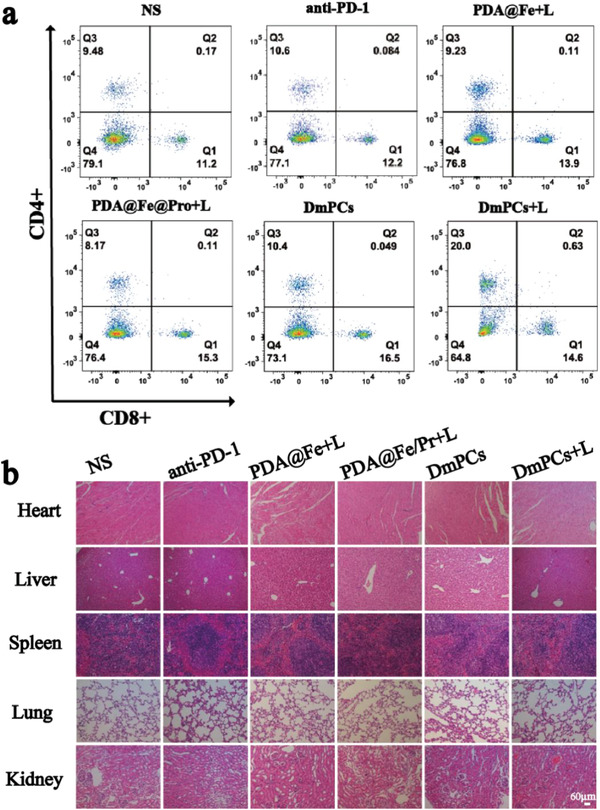
a) Flow cytometric analyses of CD4, and CD8 T cells in tumor of mice immunized by various treatments. b) H&E staining of the main organs (heart, liver, spleen, lung, and kidney) from mice in various formulations after 12 days of treatments (scale bars: 60 µm).

Cancer immunotherapy works by activating the innate immune system toward the recognition, targeting, and clearance of cancer cells. In vivo results showed that combination of PTT, as well as CDT, with DmPCs and laser irradiation and PTT‐induced immunotherapy, can effectively inhibit tumor growth. Thus, we investigated the immune response induced by DmPCs in tumor‐bearing mice. After 12 days of treatment, tumors were collected to assess the activation of immune effector cells. For that, we analyzed the level of tumor‐infiltrating T cells (CTL) and cytokine expression. CTL directly targets several types of cancer cells. However, immune tolerance within the tumor microenvironment may lead to CTL dysfunction and failure. Therefore, CTL quantification might reflect the effectiveness of the antitumor treatments. Using flow cytometry, we observed that, compared with other groups, there was an increase of CD4^+^ cells in DmPCs + laser (20%), which is higher than the increments from the groups treated with saline (9.48%), anti‐PD‐1 only (10.6%), PDA@Fe + laser (9.23%), PDA@Fe@Pr + laser (8.17%), and DmPCs only (10.4%) (**Figure** **7**a). There was an increase of CD8+ cells in DmPCs + laser (14.6%) compared to the groups treated with saline control group (11.2%) (Figure 7a). Therefore, we propose that a synergistic action between PTT and CDT could further promote the expression of CD4^+^, thus reducing CTL depletion. The above results confirmed that CD4^+^ and T cells were reactivated by our combined anti‐cancer immunotherapy and the immune response was improved by combination of various modality therapies and immunotherapy, resulting in a significant increase in anti‐tumor treatment efficiency.

We further performed histological analysis of the main organs by H&E staining. We observed no evidence of inflammation and exudation or other pathological lesions in the heart, liver, spleen, lung, and kidney, thus demonstrating the low toxicity of DmPCs to normal tissues (Figure 7b). Therefore, we propose that DmPCs nanoparticles are a potential candidate nanomedicine for combined anti‐cancer immunotherapy.

Tumor necrosis factor *α* (TNF‐*α*) is a potent inflammatory cytokine with anti‐tumor effects while interleukin 10 (IL‐10) is a potent anti‐inflammatory cytokine and is capable of suppressing the cellular immune response. So we detected the levels of TNF‐*α* and IL‐10 in the tumor tissues. The results indicated that TNF‐*α* was significantly increased in the DmPCs+L group, while IL‐10 was significantly reduced in the DmPCs+L group compared with the control group. Therefore, these results further illustrated a tumor microenvironment with effective anti‐tumor immunity by the DmPCs+L group (Figure S16, Supporting Information).

## 3 Conclusion

We designed and synthesized a core–shell nanosystem of nanoparticles combined with multiple proteins (DmPCs) for multimodal tumor therapy (CDT, PTT, and immunotherapy), and real‐time tumor imaging. Our results show that DmPCs can effectively concentrate at the tumor site, and deliver structurally and functionally stable proteins. As the nanoparticles disintegrate in a weakly acidic and GSH‐containing environment, the protein is released at the tumor sites, where it can perform PTT, immunotherapy, and fluorescence imaging‐related functions. Moreover, photothermal conversion properties of PDA enhance the immunotherapy properties of the generated nanoparticles. Overall, we provide a new approach for the combined use of multiple proteins and biological materials for tumor therapy.

## Conflict of Interest

The authors declare no conflict of interest.

## Supporting information

Supporting information

## Data Availability

The data that support the findings of this study are available from the corresponding author upon reasonable request.
