# Supplementary material for: Protein Binding Nanoparticles as an Integrated Platform for Cancer Diagnosis and Treatment
Source: Adv Sci (Weinh). 2022 Aug 18;9(29):2202453. doi: 10.1002/advs.202202453 (PMC9561793; doi:10.1002/advs.202202453)
Supplement: Supplementary file 1 — Supporting information [file ADVS-9-2202453-s001.pdf]

ADVANCED SCIENCE
Open Access

Supporting Information

for *Adv. Sci.*, DOI 10.1002/advs.202202453

Protein Binding Nanoparticles as an Integrated Platform for Cancer Diagnosis and Treatment

Xuemei Wang, Shengbo Li, Siqi Wang, Shuo Zheng, Zhenbing Chen and Heng Song**

Supporting information

Protein binding nanoparticles as an integrated platform for cancer diagnosis and treatment

Xuemei Wang[#], *Shengbo Li*[#], *Siqi Wang, Shuo Zheng, Zhenbing Chen*[*], *Heng Song* [*]

X.M.Wang, S.Q.Wang, S.Zheng, Prof.H.Song
College of Chemistry & Molecular Science, Key Laboratory of Combinatorial Biosynthesis and Drug Discovery, Wuhan University, Wuhan 430072, China
E-mail: hengsong@whu.edu.cn
S.B.Li, Prof.Z.B.Chen
Department of Hand Surgery, Union Hospital, Tongji Medical College, Huazhong University of Science and Technology, Wuhan 430022, China
E-mail: zbchen@hust.edu.cn

Materials

Dopamine, ammonia, chloroform were purchased from Sinopharm Chemical Reagent. HEPES buffer (2-[4-(2-Hydroxyethyl)-1-piperazinyl] ethanesulfonicacid), Ferric oxide (Fe_2O_3) and 1-(3-Dimethylaminopropyl)-3-ethylcarbodiimide Hydrochloride (EDCI) were purchased from Aladdin. L(-)-Glutathione was purchased from Shanghai Dipper. 5,5'-Dithiobis (2-nitrobenzoic acid) (DTNB), rhodamine B, Cholesterol and Titanium sulfate ($TiSO_4$) were purchased from Meryer. Tris(hydroxymethyl) aminomethane (Tris), Hydrogen peroxide(H_2O_2) sodium chloride (NaCl), potassium chloride (KCl), disodium hydrogen phosphate (Na_2HPO_4), potassium dihydrogen phosphate (KH_2PO_4) and sulfuric acid (H_2SO_4) purchased from Shanghai HUSHI. 2',7'-dichlorofluorescin diacetate (DCFH-DA) purchased from Beijing Pulilai Gene Technology Co. DSPE-mPEG2000 were purchased from Aviator (Shanghai) Pharmaceutical Technology Co., The mini protein (sequence: (SNTSESF)2KFRVTQLAPKQIKE-COOH) was purchased from Gill Bio (Shanghai, China)

Cell culture

B16 was purchased from fenghui biological Co. Ltd. (Hunan, China), and was cultured in Dulbecco's modified Eagle's medium (DMEM; Gibco, USA) high glucose containing 10% fetal bovine serum (FBS; HYCEZMBIO, China) and 1% penicillin/streptomycin at 37 ℃ in a humidified atmosphere of 95% air and 5% CO_2.

Animals

The C57 mice (4 – 6 weeks, female) were obtained from the Sibeifu Beijing Biotechnology Co. Ltd. All protocols were approved by the Animal Care Committee of Tongji Medical College.

Experimental Section

Preparation of Fe coated polydopamine nanospheres (PDA@Fe)

2 mL aqueous ammonia solution (2 mL, NH_4OH, 28-30%) was washed with 40 mL ethanol and 90 mL deionized water. 7.5 mg of Fe_2O_3 was added to the solvent and fully dissolved. Dissolve dopamine hydrochloride (0.5 g) in deionized water (10 mL), and then inject it into the above mixed solution. The color of the solution immediately turns light yellow and gradually turns dark brown. The reaction was subjected to magnetic stirring at room temperature for 24 hours. Obtain by centrifugation (10000 rpm, 10 min and at room temperature) and washed 3 times with water. Freeze drying.

Preparation of PDA@Fe@Pr

Weight 10mg dopamine nanospheres and disperse them to 10 mL Tris aqueous solution with pH 7.4. Disperse them evenly by ultrasound and add 5 mg EDCI. After magnetic stirring, add 2.5 mg mCherry protein, 2.5 mg anti-PD-1 protein and 2.5 mg glucose oxidase. It was obtained by centrifugation (10000 rpm, 10 min and at 4℃) after reaction at 4℃ for 24 h and washed with water for 3 times. Unreacted proteins were removed by washing with water through several centrifugations. Freeze drying.

Preparation of PDA@Fe@Pr/Lipo(DmPCs)

DSPE / Cholesterol liposomes were prepared by liquid membrane method:

Weight 22.32 mg (0.03 mM) of DSPE and 5.37 mg (0.01 mM) of Cholesterol, add 1 mL of chloroform to dissolve the lipid, remove chloroform by rotary evaporation, and vacuum for 30 mins. Add 10 mM HEPES buffer, stir overnights at room temperature, ultrasonic for 10 min, and filter through an aqueous filter membrane with a pore diameter of 220 nm to obtain a DSPE liposome hydration solution with a concentration of 1 mg/mL, which is stored in a refrigerator at 4℃ for a standby.

1 mg of nanocomposite was added dropwise to 1mL of liposome hydration solution prepared above under stirring. After ultrasonic dispersion for 10min at 4℃ and stirring for 12 h.

Protein expression and purification of mCherry:

Plasmid pET28a-mcherry was transformed into BL21(DE3) E. coli. A single colony was grown overnight at 37℃ in LB media (10 mL) supplemented with Kanamycin (100 μg/mL). Starter culture (10 mL) was used to inoculate 1L liquid LB supplemented with Kanamycin (100 μg/mL). Cells were then grown at 37℃ to an OD 600 = 0.5 and protein expression was induced by 1 mM IPTG at 16℃. Then after another 16 hours, cells were harvested. Afterward Cells in lysis buffer (50 mM Tris-HCl, 300 mM NaCl and 10 mM Imidazole, pH 7.0) were lysed by sonication. After centrifugation (8000rpm, 50min and at 4℃), the supernatant was loaded onto a Ni-NTA column. The column was washed with 20 mL washing buffer for twice (50 mM Tris-HCl and 300 mM NaCl, pH 7.0) and then eluted with elution buffer (20 mM Tris-HCl, 1M NaCl and 250 mM imidazole, pH 7.5). Final, mcherry protein was obtained.

Sequence of mCherry

MVSKGEEDNMAIIKEFMRFKVHMEGSVNGHEFEIEGEGEGRPYEGTQTA
KLKVTKGGPLPFAWDILSPQFMYGSKAYVKHPADIPDYLKLSFPEGFKWERV
MNFEDGGVVTVTQDSSLQDGEFIYKVKLRGTNFPSDGPVMQKKTMGWEASS
ERMYPEDGALKGEIKQRLKLKDGGHYDAEVKTTYKAKKPVQLPGAYNVNIK
LDITSHNEDYTIVEQYERAEGRHSTGGMDELYK*

Materials Characterization

TEM was acquired by JEM-2100 Plus, Field Emission Scanning Electron Microscope for Zeiss Merlin Compact

Stimulus Response Release

Put 3 mL of 1 mg/mL de-emulsified DmPCs into a dialysis bag (MWCO: 3500 kDa) and then into 200 mL of PBS buffer solution (pH 7.4, pH 6.5 and 10 mM GSH) with different pH values or components. Reactions were carried out at 37°C in a shaker at 100 rpm. Each time 500 μL of dialysate was withdrawn at the indicated time points and the iron content was determined by Inductively coupled plasma mass spectrometry （ICP-MS). Spectra of the samples were characterized by UV-vis and the morphology of the samples was tested by transmission electron microscopy (TEM).

Measure the encapsulation efficiency

The encapsulation rate of liposomes was determined by low-speed centrifugation. The unencapsulated PDA@Fe@Pr was suspended in solution and after centrifugation (2000 rpm,3 min and 4℃), these nanoparticles settled due to centrifugal force, but the liposome-encapsulated nanoparticles remained dispersed in the solution, thus achieving separation.

$$\text{encapsulation rate}=\frac{m_1\text{-}m_2}{m_1}$$

The total weight of PDA@Fe@Pr weighed is noted as m_1 and the weight of the dried precipitate after centrifugation is noted as m_2

Photothermal Capability of DmPCs

A 660 nm laser was used to excite different solutions and aqueous solutions of DmPCs with different concentrations, and the photothermal properties of DmPCs were detected at different powers. A FOTRIC 226 thermal imager was used to collect physical pictures and photothermal data. The DmPCs aqueous solution with a concentration of 1mg/mL was put into a centrifuge tube, irradiated with a 660 nm laser, continuously irradiated by the laser for 6 min, and then lowered to a certain temperature, and the temperature cycle curve was measured after heating for 6 min to characterize the stability of the material.

The photothermal conversion efficiencies (η) were measured according to the reported method[1]:

$$\eta = \frac{hs(T_{Max} - T_{Surr})}{I(1 - 10^{-A_\lambda})}$$

h is the heat transfer coefficient; s is the surface area of the container. I was the laser power density and A is the absorbance at 660 nm.

$$hs = \frac{mC_{water}}{\tau_s}$$

m is the mass of the solution containing the photoactive material, C is the specific heat capacity of the solution (Cwater = 4.2 J/(g•°C)), and τs is the associated time constant.

$$t = -\tau_s \ln(\theta)$$

θ is a dimensionless parameter, known as the driving force temperature

$$\theta = \frac{T - T_{Surr}}{T_{Max} - T_{Surr}}$$

T_{max} and T_{Surr} are the maximum steady state temperature and the environmental temperature, respectively.

The photothermal effect and photothermal conversion efficiency of DmPCs were also conducted by using the 660 nm laser for irradiation.

GOx-like enzymatic activity of DmPCs

To evaluate the GOx-like enzymatic activity, 100mM glucose were produced and mixed with DmPCs or free Gox solution, followed by measure-ment of H_2O_2 and gluconic acid concentrations in the solution. To detect H_2O_2, 24% $TiSO_4$ was first prepared in 50 mL DI water containing 8.33 mL H_2SO_4. 2.1 mL of PBS, 0.2 mL glucose solution (100 mM), and 0.2 mL DmPCs nanocomposite (500 ppm) or GOx (500 ppm) were mixed in separate tubes. The absorbance at 405 nm was measured at 30 min intervals after 100 μL of glucose-containing solution was mixed with 200 μL of $TiSO_4$.

Extracellular GSH Depletion

The PDA@Fe and DmPCs were degraded by acid, and the pH was adjusted to 7 after the supernatant was prepared. The supernatant and aqueous GSH solution (50 μg/mL) were mixed for 10min at 25℃. Then 500 μL of DTNB (50 μg/mL) was added to the solution and the absorbance was measured at 412 nm by UV-Vis spectroscopy after 5 min.

Extracellular Hydroxyl Radical Detection

Acid degraded PDA@Fe and DmPCs and supernatant was prepared and adjusted to pH 3. 280 μL of 200 μM RhB in water, each group supernatant, 200 μL of 20 mM H_2O_2 and tris-HCl (pH=3) were mixed and absorbance was measured at 550 nm using UV-vis spectroscopy after 5 min. The supernatant of acid-degraded DmPCs was adjusted to pH=7, then 5,5-Dimethyl-1-pyrroline N-oxide was added and tested for the presence of •OH using electron paramagnetic resonance.

Cell Viability Assays

Cell viability of B16 cancer cells incubated with diverse concentrations of PAD@Fe and PAD@Fe@Pr/Lipo. The cells were irradiated with or without an laser (660 nm, 0.5 W/cm^{-2}, 5 min), followed by further incubation for 24 h and test cell viability. The sample solution is configured with different concentrations, ranging from 10 to 160 μg/mL, to test the survival rate of cells in different gradients.

Intracellular Detection of ROS

In Vitro 2’7’-dichlorofluorescin diacetate (DCFH-DA) was used to evaluate the ROS production. B16 tumor cells 6-well plates were treated with DmPCs and PDA@Fe at different times (4, 8, 12 h), and stained with DCFH-DA or Hoechst for photographed by fluorescence microscopy.

Cell Internalization Assay

B16 cells were cultured in DmPCs containing medium at 37°C for 4 h. After 4 h, the culture medium was discarded and washed with PBS for 3 times. The cells were then stained with Hoeches and the slides were washed with PBS three times. Finally,

the internalization of RNs was observed by confocal laser scanning microscope (PerkinElmer UltraVIEW VoX, lsm780, Germany). Fluorescence signals from Hoeches staining were detected by excitation at 350 nm and emission at 460-480 nm. The fluorescence of mCherry protein was detected at the excitation wavelength of 561 nm and the emission wavelength of 575-625 nm.

Intracellular GSH Depletion

B16 mouse melanoma cells on 6-well plates were treated with different concentrations (DmPCs: 0, 10, 50, 100 μg/mL). After cell digestion, suspension and disruption, the supernatant was collected by centrifugation at 10,000 rpm for 10 minutes at room temperature. The supernatant was then reacted with DTNB 50 μg/mL for 5 min and detected by UV-Vis spectroscopy.

Anticancer Effects and Biosafety In Vivo

The mouse tumor model was established by subcutaneous injection of 4×10^5 B16 melanocytes into the right hind limb of C57 mice. Mice were randomly divided into seven groups (n=6) and were injected intravenously with saline, PDA@Fe, anti-PD-1, PDA@Fe@Pr, DmPCs (300 μg/mL per injection, every other day through the tail). Intravenous injection once, the treatment cycle is 12 days). The PDA@Fe, PDA@Fe@Pr and DmPCs groups were respectively irradiated with 660 nm, 0.5 W/cm^2 laser for 5 min after injection for 8 h. The body weight and tumor volume of each group of mice were measured and recorded for 12 days. After 12 days of treatment, the mice were sacrificed and their blood, tumor tissue and major organs were collected. CD4 and CD8 CTLs were determined by flow cytometry. In addition,

the tumors and main organs of mice in each group were fixed in 4% paraformaldehyde, and then stained with (H&E, Ki-67 or TUNEL) to observe the pathological changes.CD4 and CD8 were determined by flow cytometry.

Figure S1-S10

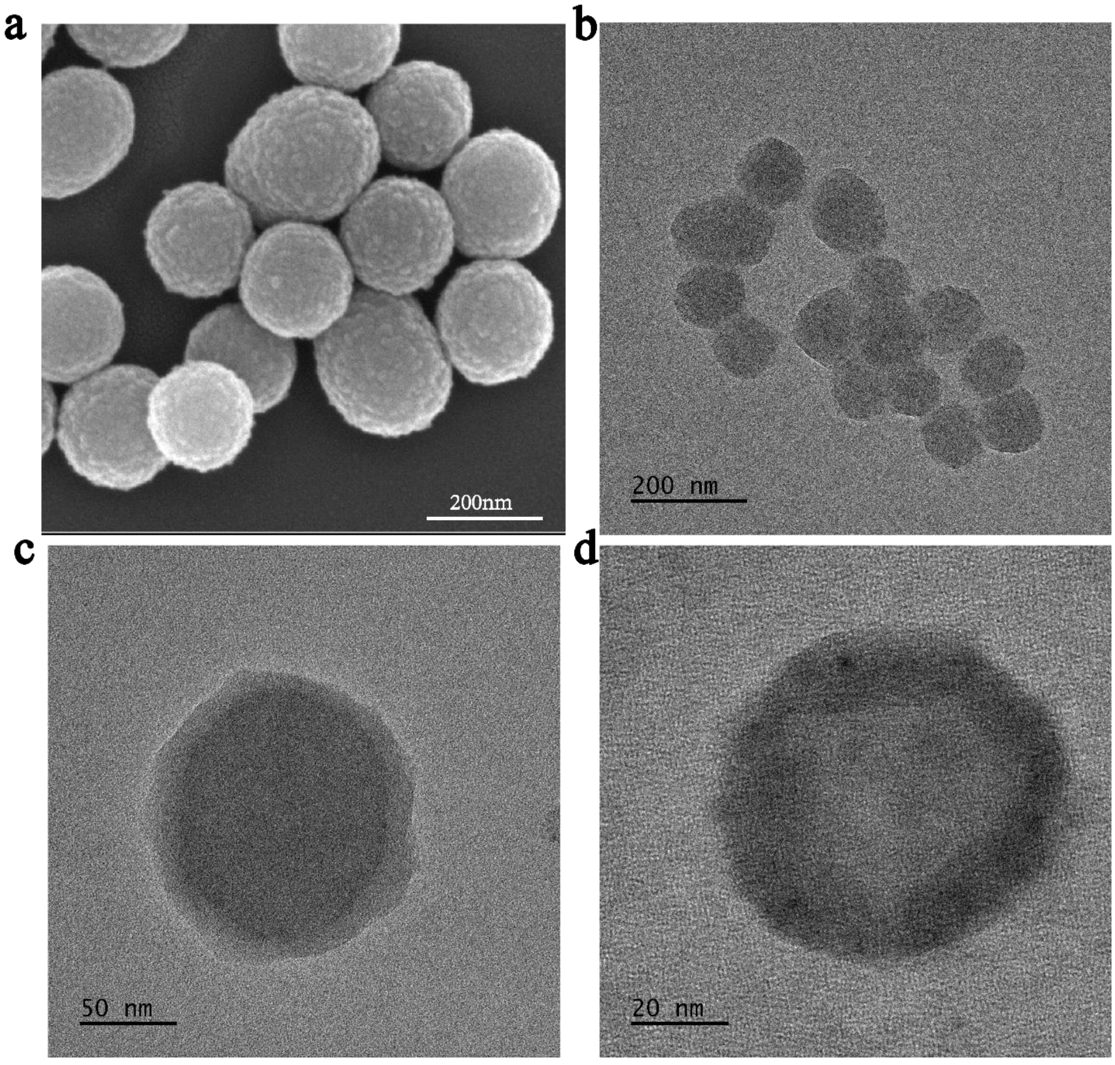

Figure S1. a) The SEM images of PDA@Fe. b) The TEM images of PDA@Fe. c) The TEM images of PDA@Fe@Pr. d) The TEM images of DmPCs.

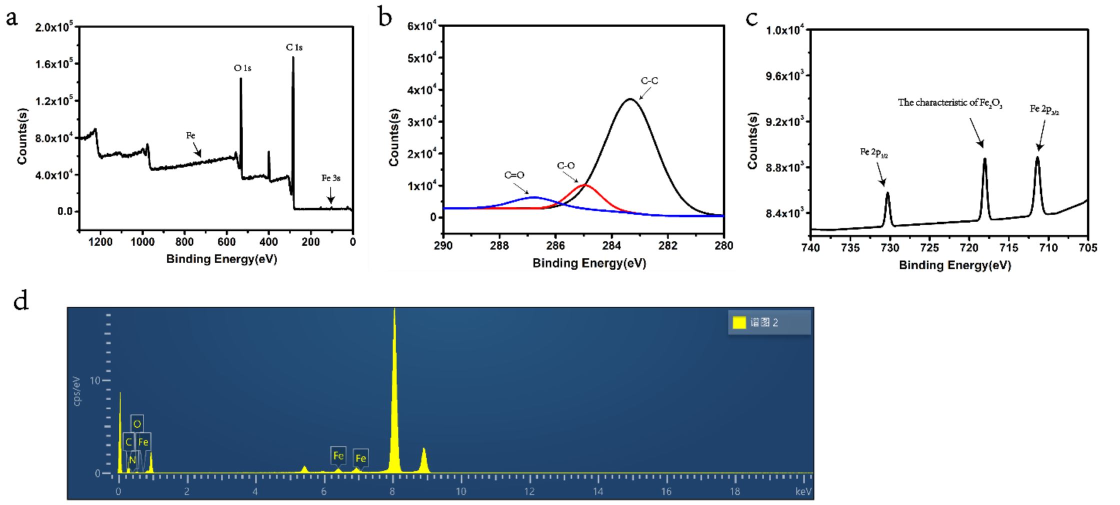

Figure S2. The XPS analysis of DmPCs: a) survey spectrum b) C1s spectrum c) Fe spectrum d) PDA@Fe TEM Energy Dispersive Spectrometer.

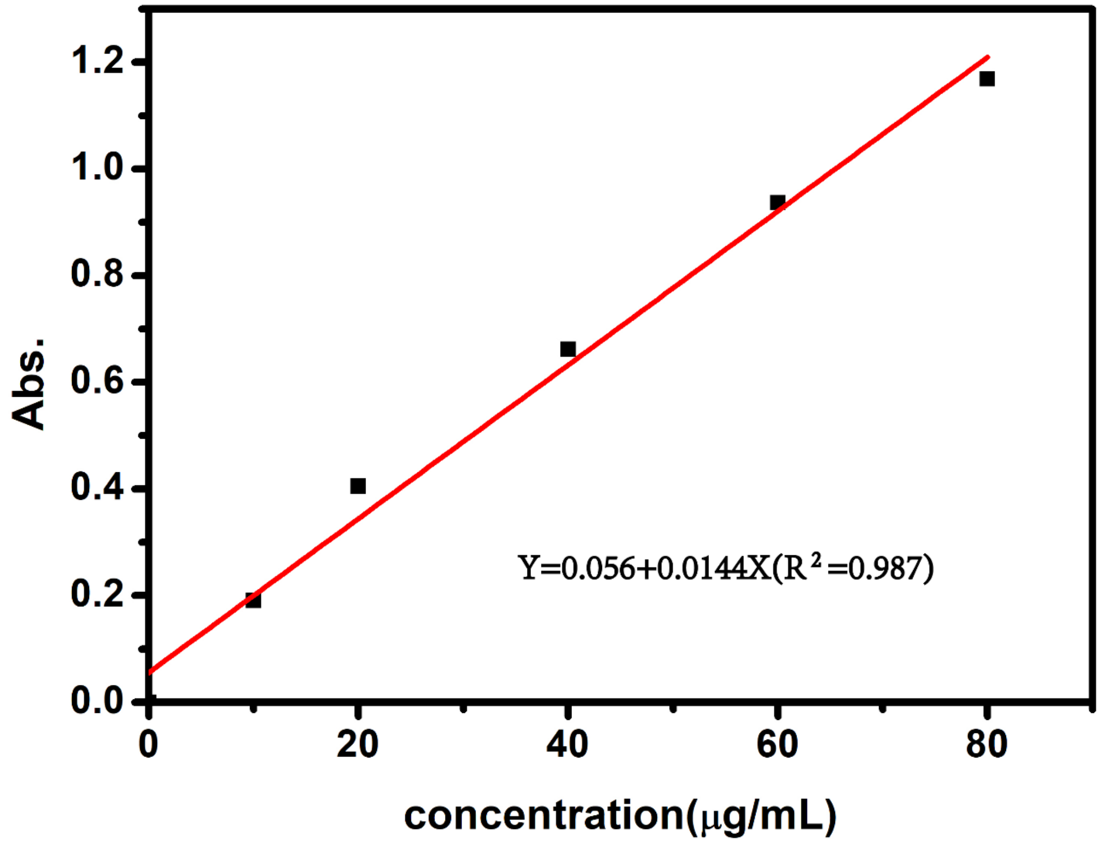

Figure S3. Fe concentration curve.

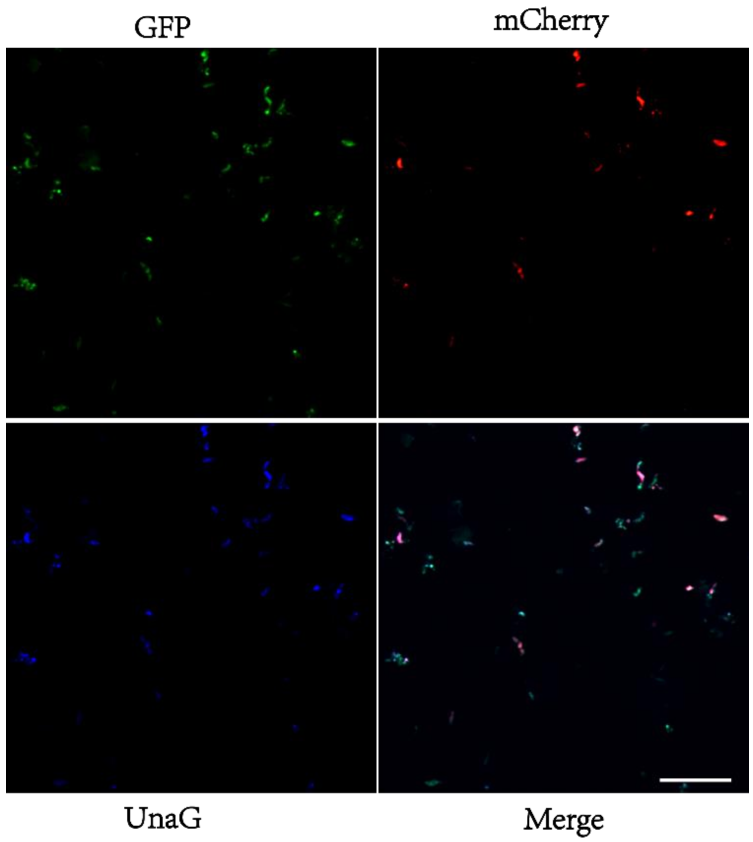

Figure S4. Fluorescence images of B16 cells costained with three proteins (Scale bars:36 μ m).

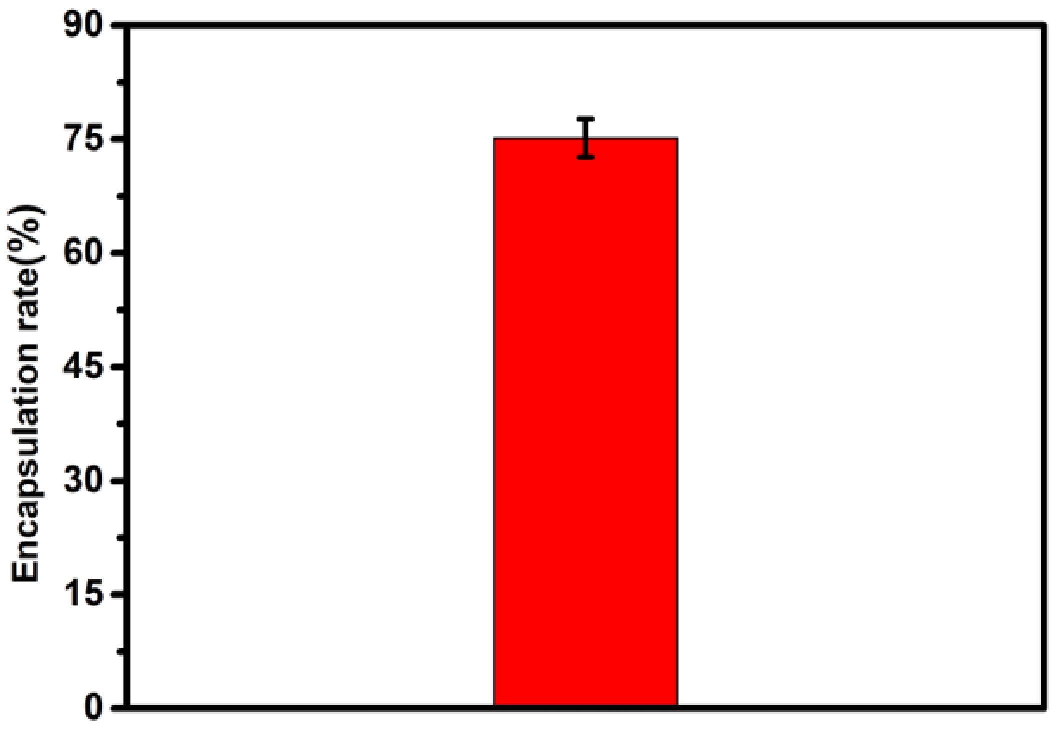

Figure S5. Encapsulation efficiency of liposomes.

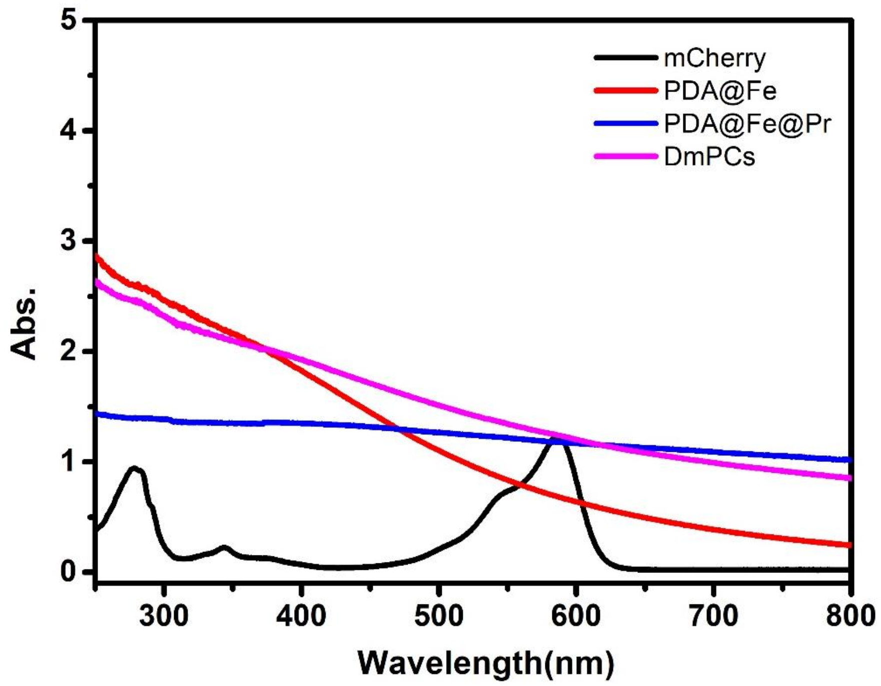

Figure S6. UV−vis absorption spectra of PDA@Fe,PDA@Fe@Pr mCherry and DmPCs

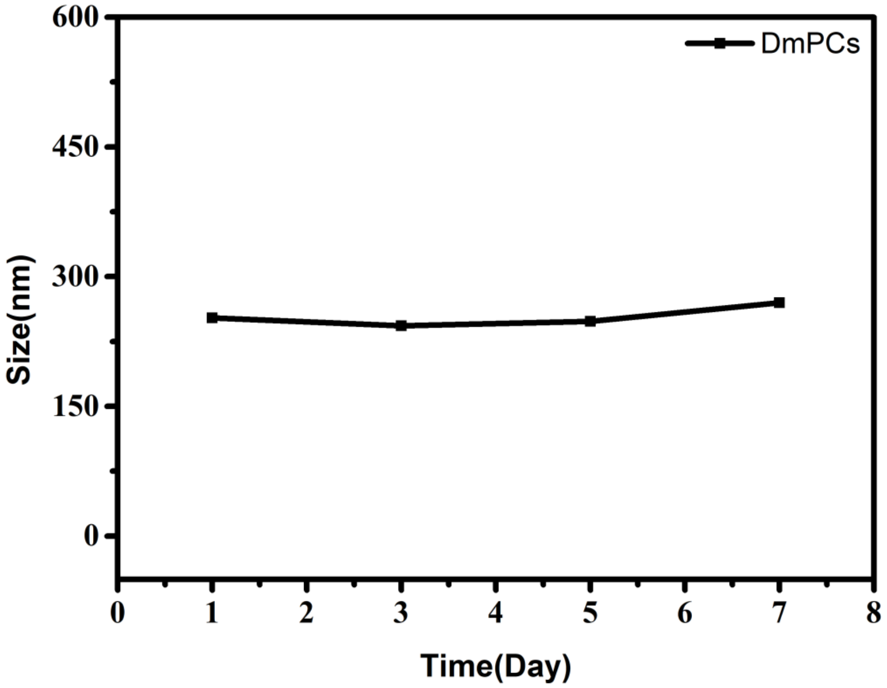

Figure S7. The long-term stability of DmPCs complexes in PBS.

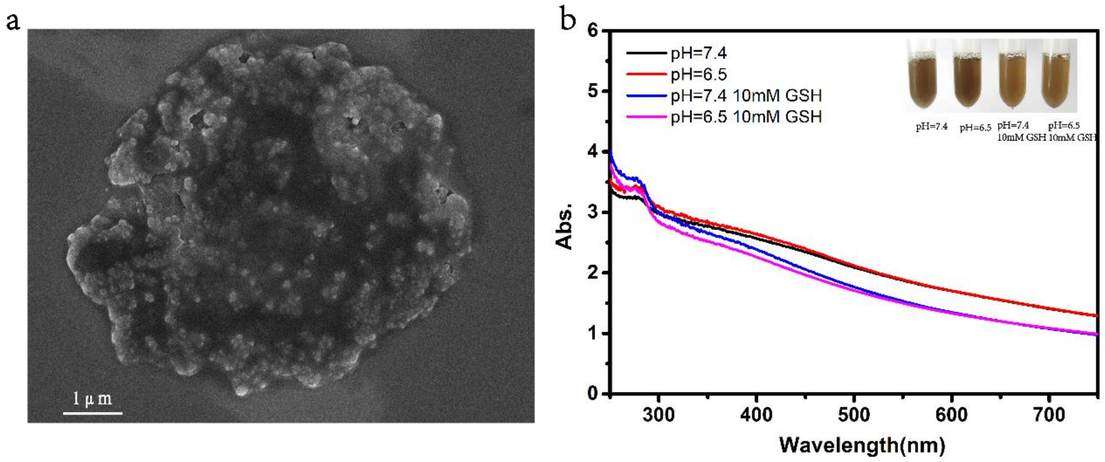

Figure S8. (a) The SEM image of DmPCs under 10mM GSH conditions at 37 ℃. (b) UV−vis absorption spectra of DmPCs (0.5mg/mL) solution after incubation in different conditions for 60 h.

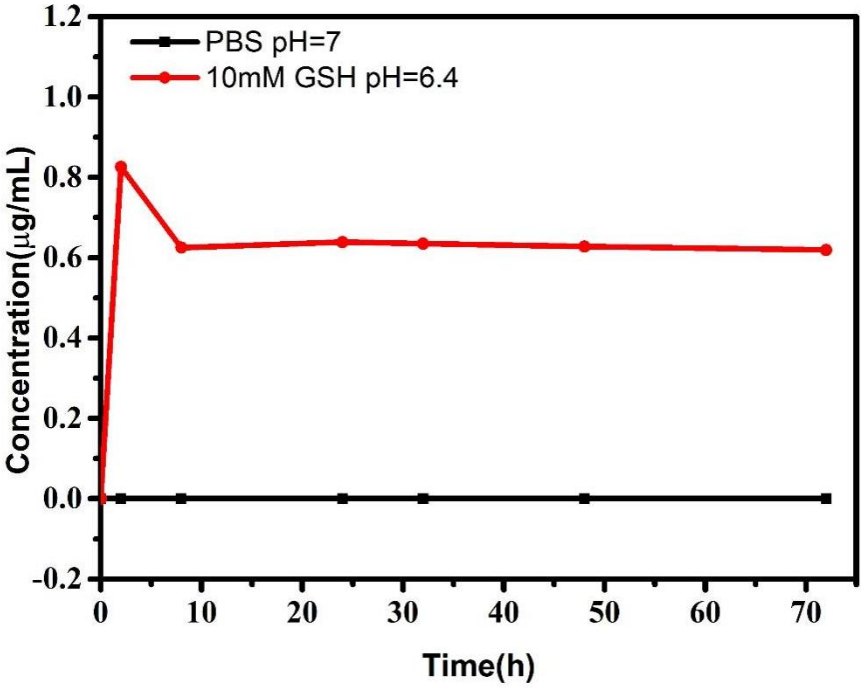

Figure S9. Fe ions release in different conditions in 10mM GSH and pH=6.5 solution.

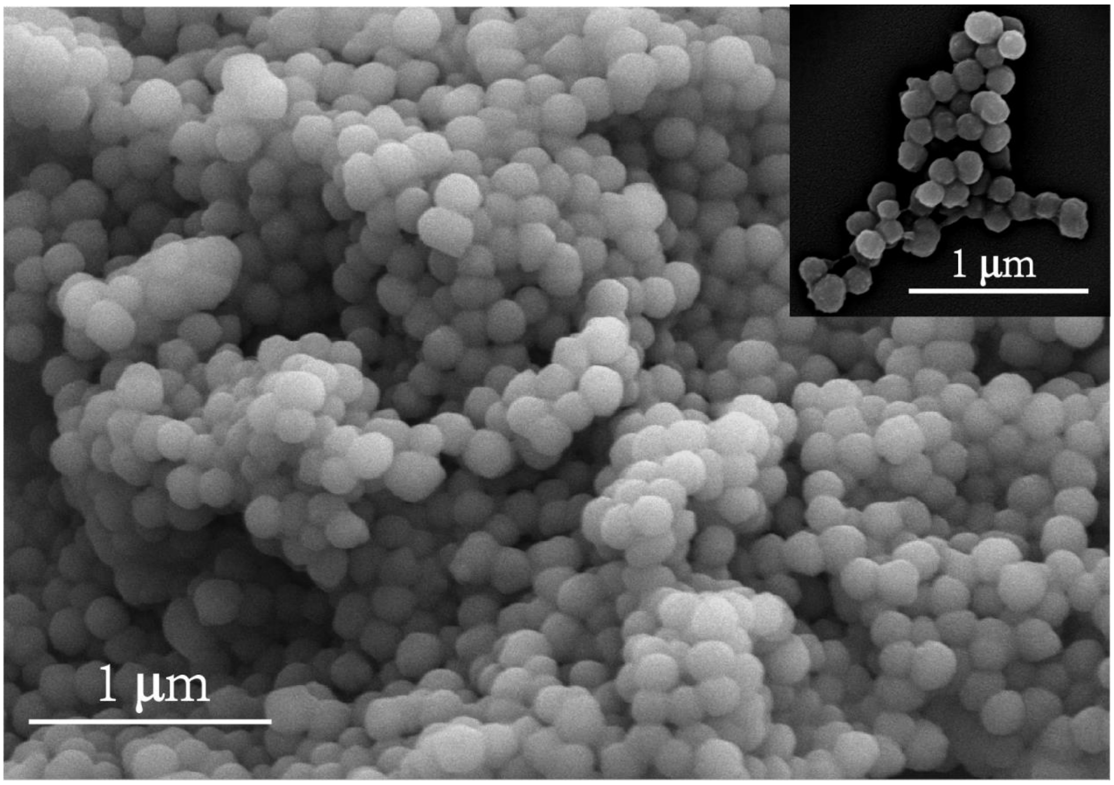

Figure S10. SEM of DmPCs under 660 nm laser (0.5 W/cm^2).

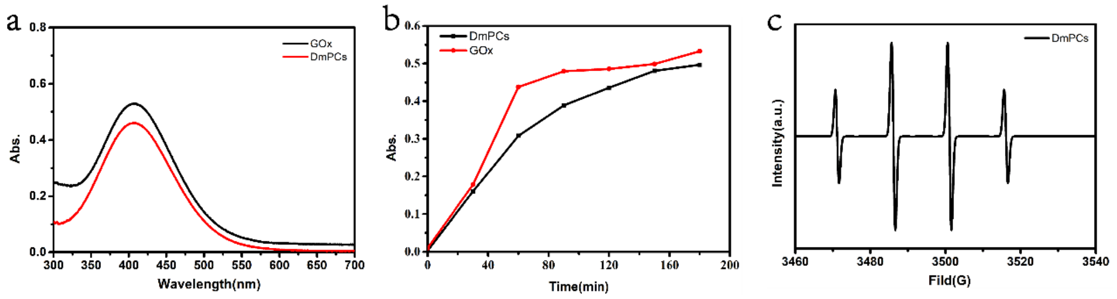

Figure S11. (a) UV–vis spectra of $TiSO_4$ solutions with 100mM glucose for 500 ppm of GOx and DmPCs. (b) Absorption intensity at fixed wavelength (λ = 405 nm) for the analysis of H_2O_2 production. (c) The EPR spectra of DmPCs and hydroxyl radical generated in the H_2O_2/DmPCs/DMPO.

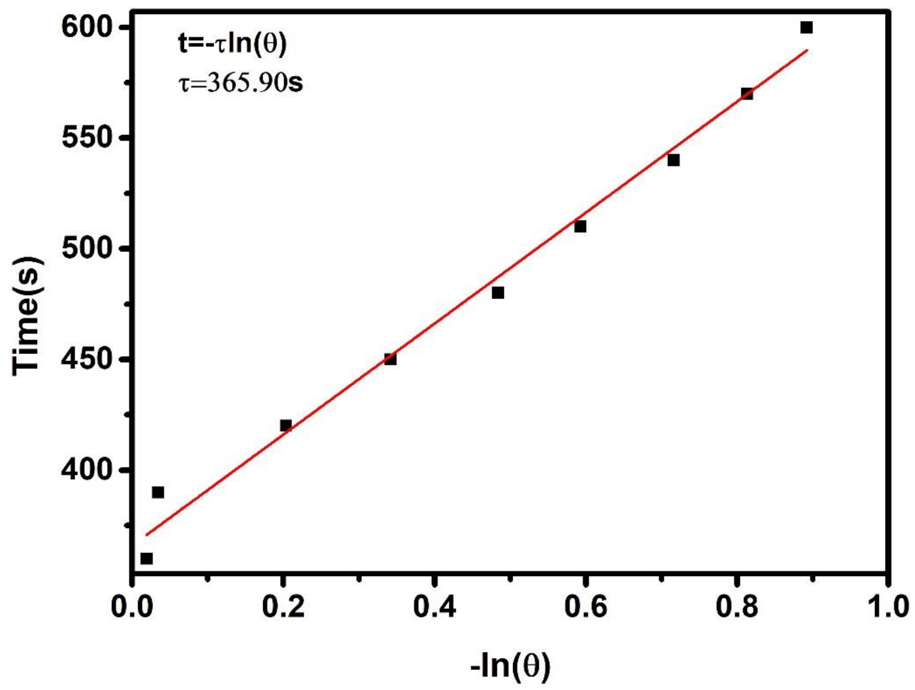

Figure S12. The time constant for DmPCs.

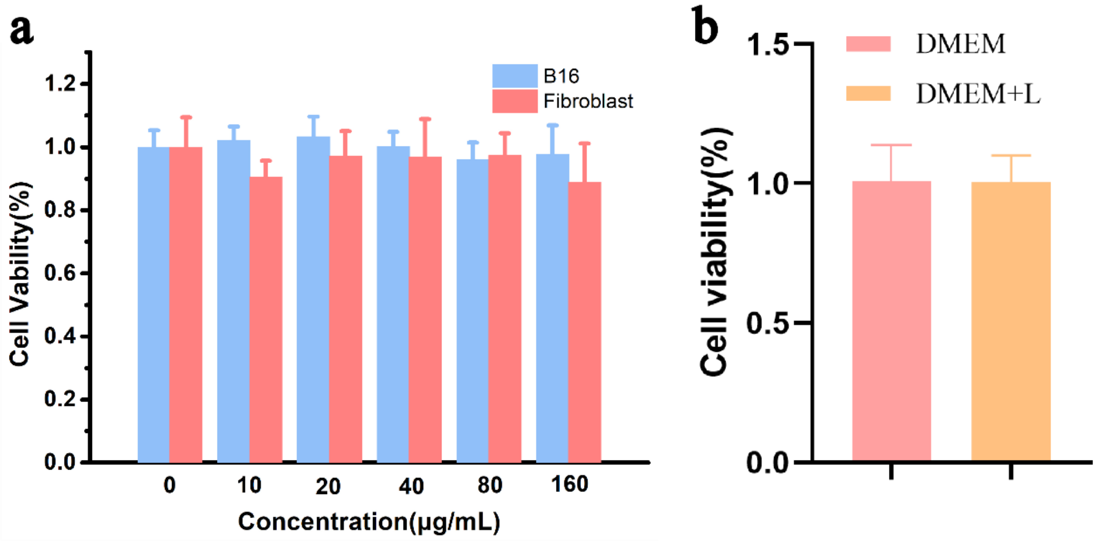

Figure S13. (a) Cell viability of B16 and Fibroblast after incubation for 24 h. (b) Cell viability of B16 were irradiated with or without a laser (660 nm, 0.5 W/cm^{-2}, 5 min), followed by further incubation for 24 h.

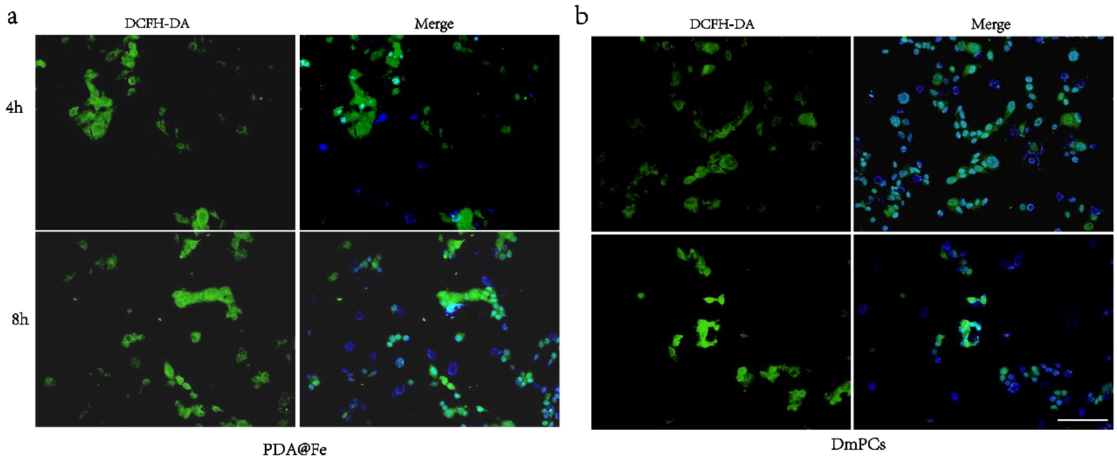

Figure S14. ROS intensity in B16 cells treated with DmPCs and PDA@Fe at different time points as examined by fluorescence microscope (Scale bars:150 μm).

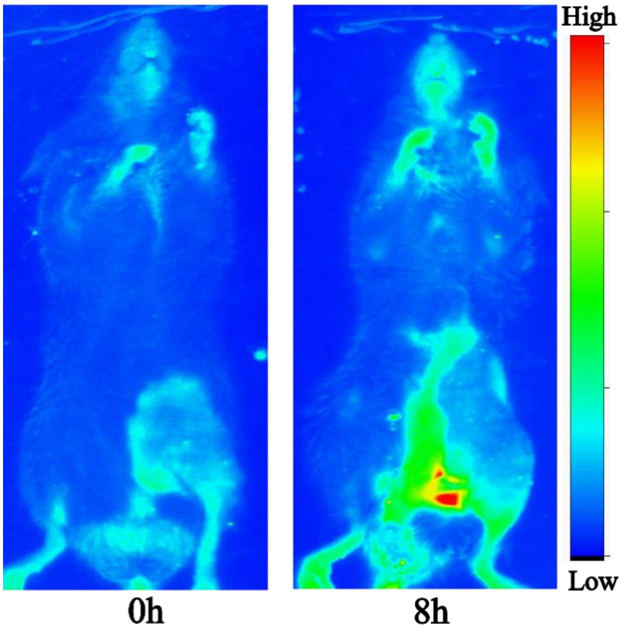

Figure S15. In vivo fluorescence images of DmPCs in B16 tumor-bearing mice.

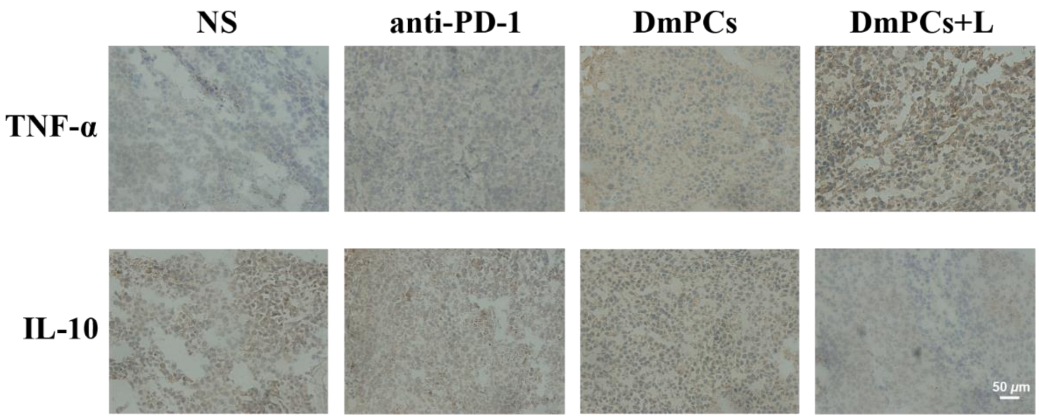

Figure S16. (a) Tumor necrosis factor α (TNF-α) staining (b) interleukin 10 (IL-10) staining of tumors in various formulations after 12 days of treatments. (scale bars: 50 μm)

Conditions	PBS with 50μg/mL (0.162 mM) GSH	B16 cells in a six-well plate
Absorbance (412 nm)	0.5283±0.064	0.14407±0.0015

Table S1. UV absorbance at 412 nm after the GSH in different conditions reacting with DNTB.

[1] D. Xi, M. Xiao, J. Cao, L. Zhao, N. Xu, S. Long, J. Fan, K. Shao, W. Sun, X. Yan, *Advanced Materials* **2020**, 32, 1907855.
